# COVID-19: our last teachable moment

**DOI:** 10.35241/emeraldopenres.13603.2

**Published:** 2020-05-21

**Authors:** Gerard Hastings

**Affiliations:** 1 Institute for Social Marketing and Health, Stirling University, Stirling, UK

**Keywords:** Covid 19, change, climate breakdown, hope

## Abstract

COVID-19 is bringing hardship and tragedy.  Health workers are having to take appalling risks; loved ones are being lost; lockdown is causing great distress.  And, as always in testing times, the disadvantaged are being hit worst. As we emerge from the shadows, the call from the vested interests, from the systems current winners, will be for a rapid return to business as usual. We must resist this; business as usual got us into this mess.

COVID-19 is trying to tell us something; we health educators and social marketers must listen, think and, above all, take action.

## Heeding the call

COVID-19 is not the first warning we have had, but it is the first one we have had to heed. There have been other epidemics (BSE, SARS, MERS, Ebola) and other financial crises (1929, 1973, 1997, 2000, 2008), but none has challenged us like COVID. Never before have we closed down our economy; stopped making, distributing, buying and consuming on a global scale. Governments have been transformed, as if by magic, from reluctant nannies into muscular interventionists. Overnight, cheap flights, pints in the pub and drives in the country have vanished; friends and family have gone off-limits. No previous calamity has so obviously touched everyone, from the Amazonian villager to the captain of industry; from the head of state to the rough sleeper: we are all threatened by the virus; we all have to self-protect; we all have to accept our individual and collective responsibilities. Those who don’t, whatever their background, are castigated; Lear jets and fat bank accounts might give you access to better outcomes ^1^, but the rich still have to obey the lockdown rules just like the rest of us ^2^. It is a rare moment of global unity and a valuable reminder of how much we have in common, how closely we depend on each other, how important our environment is to us. As a result, we have a unique opportunity to rediscover our humanity, to question our assumptions and to learn. Unique not just because we have squandered previous warnings, but also because future ones are likely to be too extreme to provide any teachable moments.

COVID-19 has got our attention. It is telling us something about the state of our health systems, which have struggled to respond. It is telling us something about how we deal with epidemics, the roll of testing and herd immunity. It is telling us something about values: that caring is precious; that life is precarious; that human beings do, after all, matter more than money. But most of all it is telling us something about the flaws in our economic system. We already know about these, of course - sweatshops, conflict minerals, congenital inequality, plastic waste are all familiar scandals - but they have somehow remained remote. Even climate breakdown, and the Intergovernmental Panel on Climate Change (IPCC)’s dire warnings have resulted in little more than foot-dragging and excuses. Certainly, they have not caused us to act: more than half the C02 in the atmosphere has been put there since the IPCC’s first report was published in 1988 ^3^.

Having got our attention, COVID-19 has also delivered up a remarkable experiment: what happens when neoliberal capitalism is put on hold? When the factories close, the supply chains fracture, the shopping stops? A study which would never have been deemed ethical or feasible heretofore has gone ahead almost unnoticed, and the data is now in. The two-month economic shut-down in China improved air quality to such an extent that 77,000 lives were saved, including those of 4,000 under-fives ^4^. This is twenty times more than were taken by the virus. Far from the cure being worse than the disease, it turns out to be far better than business as usual; switching off capitalism not only protects us from the virus, it protects us from ourselves.

Predictably perhaps, USA Today downplays the significance of the data: "At the most” it argues “it shows it's easy to overlook chronic, long-term health threats such as air pollution, and thus, harder to muster an adequate response” ^5^. Le Monde however, sees prima face evidence of systemic problems with neoliberal global capitalism ^6^, as does the French President whose March 12 address to the nation proclaimed the need to cross-examine our economic system which has been shown by COVID-19 to be so conspicuously flawed ^7^. The fact that the virus might also have negative environmental impacts, such as increasing our consumption of single-use plastics, only reinforces the need for a rethink ^8^.

The figures from China also bring a message of hope: when we stop behaving badly, things can get better fast. The air improved and lives were saved as soon as the factories stopped. In Italy the venetian canals turned back from black to blue just days after the shut-down began; in Paris birdsong became audible for the first time in generations as soon as the traffic cleared. This chimes with longer term evidence showing that the oceans can recover when adequately protected, that whale populations grow with the control of commercial hunting. Living in an era called the anthropocene brings with it an onerous burden of responsibility for planetary harm, but it also reminds us that change is possible: what we have despoiled we can reinstate.

The COVID warning is timely: we can still do something.

## What has this got to do with health education?

At this point readers of Health Education might be asking what has this got to do with me? Shouldn’t we be talking about smoking, drinking and junk food? About how to get people to take up sound, evidence-based health advice? About social distancing and handwashing? Not capitalism and the meaning of life. But these traditional public health concerns link directly to our current plight.

One of the most significant advances is public health thinking over the last forty years has been the recognition and calling out of industrial epidemics and the commercial determinants of ill-health. The realisation that, whilst smoking, drinking and junk food are individual health behaviours, they are also big businesses – very big. In a world dominated by neoliberal capitalism this makes them remarkably powerful; corporations have become some of the largest and most formidable organisations on earth. They now dwarf countries: “ *the annual revenue of each of the five largest global corporations exceeds $250 billion, more than the GDP of 75 percent of the world's nations”* ^9^, but have no democratic controls, few checks and balances and only one duty: to deliver ever greater returns to shareholders.

Their abundant wealth enables them to buy the best marketing and lobbying expertise. The history of tobacco control shows how powerful these tools are: the public health case can only be won when they are countermanded. In the UK, for example, where the last twenty years have seen the systematic removal of nearly all tobacco marketing, teen smoking has dropped to less than 5%. But in many countries tobacco marketing and smoking continue apace, and across the world marketing for everything else is expanding and, with digital, becoming ever more powerful. Shoshana Zuboff’s ^10^ forensic analysis shows how surveillance capitalism has given the marketer nuclear capability. By tracking our every keystroke, supplementing this with other sources of personal information and harnessing the resulting ‘big data’ to artificial intelligence they can determine what we know, feel and do even before we do – and manipulate us with the confidence of a puppeteer. Marketing is no longer a hit and miss mix of judgement and artistry, it is a matter of “guaranteed outcomes” and “scientific certainty”. COVID 19 has served to underline this power, as Mark Grindle ^11^ points out: “ the UK Government’s first response to coronavirus was to allow big US tech companies to centralise and mine confidential UK patients’ health data” and “the Scientific Group for Emergencies (SAGE) advising the UK Government’s response to the pandemic included those with evidenced and significant AI and data mining business interests.” Naomi Klein confirms that this power grab is very much an international phenomenon ^12^.

This dominance is coupled with inherent irresponsibility, as we in public health again know all too well. The tobacco, alcohol and food industries have long ignored, denied and down-played the health consequences of consuming their products in the relentless pursuit of profit; writing them off, with divine disregard, as ‘externalities’. What is true for health consequences is also true for all the other untoward effects of neoliberal business. Thomas Piketty’s ^13^ work has shown that inequalities are not just an unfortunate side effect of the way we do business, they are the necessary result of it. When Danny Dorling ^14^ argues the UK is now as unequal as it was in Dickens’ time, he is not pointing up an aberration, but exposing a systemic problem that is bound to get worse. It is the same with climate breakdown: it is inevitable that extractive business models predicated on perpetual growth, which treat pollution as one more externality and the planet as a dustbin, will cause ecological destruction. As Morens and colleagues, writing in the *New England Journal of Medicine*, point out, COVID-19 is just one more outcome of this *“global, human- dominated ecosystem that serves as a playground for the emergence and host-switching of animal viruses”* ^15^. Again, the virus is warning us: the epidemic is not an aberration but a symptom; not an act of God but an act of self-harm.

For we health promoters and social marketers, it is the mother of all behaviour change challenges. We have to help people rethink and rework, not just the odd unhealthy habit, but our entire way of life. As with any complex change problem, this will involve both collective and individual action.

## System change

Now is the perfect time to move upstream. Public health has never been in higher regard: appreciation of health workers and the need for greater investment in the health sector are at a premium. The benefits of prevention have been sanctified; a repeat performance is unthinkable. The inadequacy of market ideology, which undervalues the caring professions, treats hospitals like supermarkets and sees the public sector as a business opportunity, has been traduced as never before.

Furthermore, government has shown it can act. The mantras about free markets, consumer choice and perpetual growth that have been used to stymie public health progress since the industrial revolution have been decommissioned with the flick of a switch. Presidents and prime ministers who have previously genuflected before the multinationals and bowed to their neoliberal needs have suddenly shown us who is boss. The World Health Organization (WHO) has demonstrated its supremacy over the World Trade Organization (WTO): wealth may bring material benefits, but health is transcendent.

This awakening needs to be knitted into our polity. At a global level, WHO must be accorded its place at the top table, its budget guaranteed not in the gift of populist politicians. Decisions about trade, taxation, fiduciary mechanisms, fiscal policy all need to be made in the light of public health priorities. Similarly, at a national level, health ministries need greater powers to guide policy, and at a local level, decisions about planning, housing, sanitation should all be steered by public health.

But, just as the financiers and CEOs have to eat humble pie, so do the rest of us. The problems of climate breakdown go deeper than financial mismanagement or selfish business practices; they stem from human arrogance. An egotistical sense that we come above and are in charge of nature, rather than a part of it. We have forgotten that we are just one species in millions, which, because of its over-sized brain, has been able to rise to world dominance in a very short space of time. Our current parlous predicament demonstrates that our impressive processing power has not delivered up wisdom – at least to us in the wealthy North. As Richard Horton hesitantly acknowledges *“perhaps we can’t control the natural world after all. Perhaps we are not quite as dominant as we once thought”* ^16^. He is surely right, but somewhat late to the realisation; this is precisely what native Americans and countless other indigenous peoples have tried to tell us for centuries even as we worked systematically to exterminate them. Horton continues “ *If Covid-19 eventually imbues human beings with some humility, it’s possible that we will, after all, be receptive to the lessons of this lethal pandemic.”* I hope he is right again. Whatever world polity emerges from this catastrophe it surely needs to put the natural world in its rightful and pivotal place. And this time it should be informed by indigenous peoples; as Arundhati Roy succinctly reminds us: *“the people who created the crisis will not be the ones that come up with a solution”* ^17^.

## Individual change

Vital though systemic change is, the individual cannot be let off the hook: in the final analysis, systems are made up of people. And neoliberal capitalism is, in one sense, remarkably benign. There is no physical compulsion to join the party: the Stasi won’t give you a 3am visit for not using Amazon; there are no jackboots on the stairs if you omit to update your Tesco Clubcard; no one is hooded and beaten for not joining Facebook. We do all these things voluntarily. Indeed, we pay for the privilege, and in the process make a gift of our personal data so that our fealty can be marshalled even more effectively in the future. We don’t just succumb, we collaborate. We don’t even have the decency to be ashamed: we wear their logos, boast about our sweatshop bargains and inform on our friends and family with social media.

The antidote is agency ^18^. The most important job of health educators and social marketers is to enable critical thinking; to raise awareness of our predicament; to reveal the chicanery of marketing (as has been done so effectively with tobacco); to remind people that, even in the maw of surveillance capitalism, they have a choice: they can refuse their consent. This may sound naïve, even unethical: how can I correct the failings of Exxon Mobil? Against the might of Zuckerberg my actions will always be puny; this is just victim blaming…

There are three responses to this. The first is Greta Thunberg: small, disempowered individuals can make a difference. The second is philosophical, it concerns what it is to be human. The ability to make judgements, to think for ourselves, to retain a sense of control over our fate – however tenuous - are qualities that define us as people. Without them we become subordinate to the decision making and priorities of others; we cease to exist. Primo Levi ^19^ argued passionately that the Nazi camps were designed to dehumanise their victims and so facilitate annihilation, and that the final privation was to take away any sense of agency. You had to hold on to this, he said, and you could do so - by refusing your consent. Empowerment, taking responsibility, having a say in your own fate – these are not impositions, they are human rights.

The third answer is practical: if not me, who? If not now, when? As Theodor Geisel says at the end of his ecological fable the Lorax, *"unless someone like you cares a whole awful lot, nothing is going to get better. It's not.”* ^20^ We health educators and social marketers need to help people to care more; to think critically; to exercise their human rights. This is our job. And if Dr Seuss seems a bit whimsical, let us back him up with Percy Bysshe Shelley’s muscular reminder from the Mask of Anarchy: *“we are many, they are few”* ^21^. We not only have the wherewithal to bring about change, we have the power.

## Competitive analysis

Nothing will happen without resistance; indeed, as the ‘scream test’ has it, if there is no push-back you are not doing it right. The current vested interests will resist change except that in their own interests. This is already happening. The car and fossil fuel interests have gained billions in subsidies along with reduced ecological regulation; single-use plastics are being boosted for their (completely unproven) COVID prevention qualities; airlines are being bailed out. Thus, corporate lobbying is gathering momentum as big business sees an opportunity to benefit from the crisis.

We need to be promoting alternative ideas and policies, which put people and the planet, not profit, first. Here are some initial suggestions:

- *Bring the tech industry back into public ownership.* The internet is an amazing and potentially liberating invention; the Berners-Lee vision of a system that can connect humankind and enable everyone to participate in building a progressive and egalitarian society is admirable, and just about, still alive. It will not happen in private hands: the internet provides the highways and byways of the modern era; it is the 21 ^st^ century equivalent of the commons and needs to be rescued from enclosure.
- *Sanitise commercial marketing.* Henceforth the need to be truthful shouldn’t be a fanciful slogan ^22^, but a mandatory requirement. The French Loi Evin has demanded this of alcohol advertisers for a generation; the same discipline should be applied to all advertising. Marketing should be there for one purpose only: to help consumers make better informed decisions – better for them and the planet; emotional appeals, celebrity endorsements, branding and others forms of Maddison Avenue manipulation only hinder this.
- *Extend globalisation.* In an interconnected world, solutions have to be global. Our current problems are not caused by globalisation, just the inadequate and narrowly construed version of it focusing on economics and business which currently predominates. This needs to be broadened to take in much wider human and planetary needs; in particular, public health should be a key driver of decision making.
- *Re-engineer the corporation to compel responsibility for all its actions.* There shalt be no more externalities: every part of the business transaction has to be put on the balance sheet and opened to public scrutiny. The Green New Deal is a welcome move in this direction.
- *Fundamentally reassess our geopolitical system.* Humankind needs to come together for a fundamental rethink, not in a Davos style meeting of the usual corporate suspects, but a much wider gathering of people of all backgrounds – sociologists, ethicists, indigenous groups, environmentalists, poets, health promoters, artists - to discuss how we can share the planet harmoniously both with each other and the rest of the natural world.

## A time for action

This will call for great courage and monumental effort: the forces of neoliberalism will use every weapon at their disposal to retain their advantage. But it is our job to join, to lead, the charge for change. And when things get tough, as they surely will, remember that when global capitalism faltered the air got sweeter, the canals got cleaner and the birds sang louder.

## Footnotes

^1^ Deaths involving COVID-19 by local area and socioeconomic deprivation: deaths occurring between 1 March and 17 April 2020. Office of National Statistics. 2020. Reference Source

^2^ Willsher K (2020) Police block private jaunt to Côte d’Azur, The Guardian 11 Apr http://guardian.newspaperdirect.com/epaper/viewer.aspx

^3^ Wallace-Wells D (2019). *The Uninhabitable Earth*. Penguin Random House. ISBN 9780525576709

^4^ https://eu.usatoday.com/story/news/nation/2020/03/17/coronavirus-could-be-saving-lives-pollution-carbon-emissions/5066718002/

^5^ Ibid

^6^ Lambert R and Rimbert P (2020) Jusqu’à la Prochaine Fin de Monde, Le Monde Diplomatique Avril

^7^ Ibid p21

^8^ https://www.weforum.org/agenda/2020/05/plastic-pollution-waste-pandemic-covid19-coronavirus-recycling-sustainability/

^9^ Allen White, “The Missing Third Party: Corporations and the Social Contract,” GreenBiz, February 22, 2018, www.greenbiz.com/article/missing-third-party-corporations-and-new-social-contract.

^10^ Zuboff S (2019) *The Age of Surveillance Capitalism* Profile ISBN 13: 9781781256848

^11^ See review 1

^12^ https://www.theguardian.com/news/2020/may/13/naomi-klein-how-big-tech-plans-to-profit-from-coronavirus-pandemic

^13^ Piketty T (2013) *Capital in the Twenty-First Century* Harvard University Press ISBN 978-0674430006

^14^ Dorling D. Injustice: why social inequality persists. Bristol: Policy Press; 2012.

^15^ Morens David M, Daszak P and Taubenberger K (2020) Escaping Pandora’s Box — Another Novel Coronavirus, The New England Journal of Medicine p 1293 Downloaded from nejm.org on April 7, 2020

^16^ Horton R (2020) Covid-19 is the greatest science policy failure in a generation, The Guardian, April 10

^17^ Gupta, http://www.guardian.co.uk/world/2011/nov/30/arundhati-roy-interview

^18^ Hastings, T M (2011) A job worth doing? Reinterpreting control, resistance and everyday forms of coping with call centre work in Glasgow. PhD thesis, University of Glasgow

^19^ Levi P. If this is a man. London: Penguin Books, 1979, p. 47.

^20^ The Lorax, Random House, ISBN 0-394-82337-0

^21^ The Mask of Anarchy was written by Percy Bysshe Shelly in 1819 in the aftermath of the Peterloo Massacre

^22^ The Advertising Standards Authority, the UK’s independent regulator of advertising across all media boasts that it has been keeping advertising legal, decent, honest and truthful for 50 years, conveniently forgetting such consummate liars as the Marlboro Cowboy, the Carling Starlings and Colonel Sanders
