# Supplementary material for: COVID-19: our last teachable moment
Source: Emerald Open Res. 2020 May 21;2:20. doi: 10.35241/emeraldopenres.13603.2 (PMC7237165; doi:10.35241/emeraldopenres.13603.2)
Supplement: Peer review history

# Peer review: Reviewer response for version 1

**DOI:** 10.21956/emeraldopenres.14674.r26776

**Published:** 2020-05-14

**Authors:** Timo Dietrich

**Affiliations:** 1 Griffith Business School, Griffith University, Brisbane, Qld, Australia

- Is the topic of the opinion article discussed accurately in the context of the current literature?

Yes it is. A very enjoyable and though-provoking piece hopefully becomes a wake up call for many more decision makers as they are being pushed (by the economic force) to return to business as usual.

- Are all factual statements correct and adequately supported by citations?

Yes.

- I suggest adding the 1973 oil crisis to the listings of major financial shocks.
- I suggest revising “fat bank accounts provide no privileges” as I argue they would, particularly so during a pandemic. Perhaps a bit more critical discussions could be added to highlight this.
- Another site to lives saved and environment protected angle could be provided when stating that “ *The two-month economic shut-down in China improved air quality to such an extent that 4,000 lives were saved, including those of 4000 under-fives*”. We have had a surge in single-use plastic pollution which may arguably be causing more damage the improvement in air pollution? See for example: https://www.weforum.org/agenda/2020/05/plastic-pollution-waste-pandemic-covid19-coronavirus-recycling-sustainability/

- Are arguments sufficiently supported by evidence from the published literature?

Yes, very adequate for this opinion piece.

- Are the conclusions drawn balanced and justified on the basis of the presented arguments?

Yes. Our entire way of life has to change and health educators and social marketers are playing an important part in that. As with any complex change problem, this will involve both collective and individual action and the author provides a clear roadmap (particularly for the individual change section) in last pages of this article. Although more practical guidance around how systematic change can occur during and following COVID19 could be provided here to help social marketers and health educators better understand their role to influence upstream policy (even by just linking to some strong practical applications of upstream work). I was wondering if the flow would improve if the individual change is discussed before the system change and then finish with the competitive analysis (and perhaps change competitive analysis to “roadmap to change”) and merge the last “action” section into that roadmap section as well?

Is the topic of the opinion article discussed accurately in the context of the current literature?

Yes

Are arguments sufficiently supported by evidence from the published literature?

Yes

Are all factual statements correct and adequately supported by citations?

Yes

Are the conclusions drawn balanced and justified on the basis of the presented arguments?

Yes

Reviewer Expertise:

Social Marketing | Engagement | Marketing for Good | Change

I confirm that I have read this submission and believe that I have an appropriate level of expertise to confirm that it is of an acceptable scientific standard.

# Peer review: Reviewer response for version 1

**DOI:** 10.21956/emeraldopenres.14674.r26773

**Published:** 2020-05-11

**Authors:** Mark Grindle

**Affiliations:** 1 University of Highlands and Islands, Inverness, UK

Hastings’ article points to an ironic upside to the coronavirus pandemic, which is that Lockdown has thrown into relief the benefits that result when the march of neoliberal capitalism is put on hold: overall improved human and environmental health. It also suggests that the most disadvantaged are those who are being hardest hit by coronavirus. If we all realise our agency, Hastings argues, we can resist his predicted clarion call from ‘the system’s current winners’ for a rapid return to the same ‘business as usual’ that caused and reinforces socioeconomic inequalities in the first place.

 The article is well-argued. But timely as its argument may be, the global response to Covid-19 is fast-developing. We might then update the article with new evidence. We now know that the most disadvantaged individuals in England and Wales are 50% more likely to die from the virus than those who are better off. ^1^

 The article is well cited. Hastings’ citation of Zuboff’s ‘Surveillance Capitalism’ is entirely appropriate. Data mining gives corporate marketers and governments scientific certainty with which to manipulate health and consumption behaviours. Data is the new oil; and you don’t acquire valuable resources without mining them. When that resource is data, mining means the monitoring and surveillance of large populations.

 We might note then that the UK Government’s first response to coronavirus was to allow big US tech companies to centralise and mine confidential UK patients’ health data ^2^. And we may note that the Scientific Group for Emergencies (SAGE) advising the UK Government’s response to the pandemic included those with evidenced and significant AI and data mining business interests. ^3^

 The one consistent priority of the UK Government and its advisors since before lockdown has not been testing, tracing and isolating or the sufficient provision of PPE for vulnerable frontline NHS and care home workers. It has been to develop and release a ‘tracing’ smartphone app whose purpose is to monitor citizens’ movements, contacts, behaviours and yes - health data relating to Covid-19 - at a national level. It further centralises and conveniently packages valuable health data.

 Yes, we all want to get back to work – and safely. But when the passport back to work for those already disadvantaged is to buy a smartphone, pay for the data contract, download the app and pay the ongoing data charges - with the psychological stress that inevitably entails - we have to ask, who really stands to profit?

 The desires of vested interests for ‘business as usual’ is then not something that *will *return, as Hastings argues. Even at a time of a national health emergency they’ve not been curtailed. It’s going to be hard to resist not just the return to business as usual but a new world order. And as we witness the mixed messaging emanating from Whitehall about ending the lockdown - and the anxiety that that is causing – it is unlikely to be the disadvantaged who will benefit from it. But if we do learn who Covid-19 and business as usual most disadvantages, we can exercise our human agency to say no.

Is the topic of the opinion article discussed accurately in the context of the current literature?

Yes

Are arguments sufficiently supported by evidence from the published literature?

Yes

Are all factual statements correct and adequately supported by citations?

Yes

Are the conclusions drawn balanced and justified on the basis of the presented arguments?

Yes

Reviewer Expertise:

Digital Health; Digital Platforms to address mental health in frontline staff during Covid-19, Digital Media Marketing, Digital Storytelling for health behaviour change and therapeutic interventions; Narrative Science

I confirm that I have read this submission and believe that I have an appropriate level of expertise to confirm that it is of an acceptable scientific standard.

## References

[1] Deaths involving COVID-19 by local area and socioeconomic deprivation: deaths occurring between 1 March and 17 April 2020. *Office of National Statistics*.2020; Reference source

[2] : UK government using confidential patient data in coronavirus response. *The Guardian*.2020; Reference source

[3] : No 10 advisers at Sage meetings: key questions that need answering. *The Guardian*.2020; Reference source
